# Antimicrobial resistance in *Neisseria gonorrhoeae* in China: a meta-analysis

**DOI:** 10.1186/s12879-016-1435-0

**Published:** 2016-03-03

**Authors:** Yawen Chen, Yanhong Gong, Tingting Yang, Xingyue Song, Jing Li, Yong Gan, Xiaoxv Yin, Zuxun Lu

**Affiliations:** Department of Social Medicine and Health Management, School of Public Health Tongji Medical College, Huazhong University of Science and Technology, Wuhan, Hubei China

**Keywords:** *Neisseria gonorrhoeae*, Gonorrhea, Antimicrobial, Resistance, Susceptibility

## Abstract

**Background:**

*Neisseria gonorrhoeae* (*N. gonorrhoeae*) resistance to antimicrobial has been a major concern in China, and epidemiological data on *N. gonorrhoeae* resistance are not well understood. This meta-analysis was aimed at summarizing the evidence on *N. gonorrhoeae* resistance to penicillin, tetracycline, ciprofloxacin, ceftriaxone and spectinomycin in China.

**Methods:**

Two researchers independently searched five databases to identify studies on *N. gonorrhoeae* resistance to antimicrobials from the databases’ inception to November 7, 2014. A random-effects model was used to estimate the antimicrobial resistance rates and their corresponding 95 % confidence intervals (CIs). Publication bias was assessed with the Begg rank correlation test and the Egger test.

**Results:**

We included 127 studies in our synthesis reporting antimicrobial resistance. Our analyses demonstrated that *N. gonorrhoeae* resistance to penicillin and tetracycline respectively increased from 74.41 % (95 % CI: 64.1–84.7 %) and 68.3 % (95 % CI: 58.7–78.0 %) in 2000 to 84.2 % (95 % CI: 79.7–88.8 %) and 82.4 % (95 % CI: 79.9–84.7 %) in 2012. *N. gonorrhoeae* resistance to ciprofloxacin experienced a steady increase from 12.7 % (95 % CI, 8.6–16.7 %) in 1995 and reached 93.8 % (95 % CI: 91.9–95.7 %) in 2003. *N. gonorrhoeae* resistance to ceftriaxone was 1.7 % (95 % CI: 0.5–5.7 %) before 1995 and 0.5 % (95 % CI: 0.2–1.4 %) in 2012, and *N. gonorrhoeae* resistance to spectinomycin was less than 2 % from 1995 to 2012.

**Conclusions:**

*N. gonorrhoeae* resistance rates to penicillin, tetracycline and ciprofloxacin were high in China. Ceftriaxone and spectinomycin remained effective therapy for the treatment of gonorrhea. It is essential to strengthen *N. gonorrhoeae* resistance surveillance and update treatment guidelines timely.

**Electronic supplementary material:**

The online version of this article (doi:10.1186/s12879-016-1435-0) contains supplementary material, which is available to authorized users.

## Background

*Neisseria gonorrhoeae* (*N. gonorrhoeae*) is the causative agent of gonorrhea, which is one of the most prevalent sexually transmitted diseases (STD) and causes male urethritis, female endocervicitis, and severe reproductive complications. It also increases the transmission of human immunodeficiency virus, resulting in substantial morbidity and socioeconomic consequences [1]. The World Health Organization (WHO) estimated 106 million new cases of gonorrhea worldwide in 2008 [2, 3]. In China, patients with *N. gonorrhoeae* visited STD clinics or hospital outpatient departments for treatment and 95,263 cases of gonorrhea were reported in 2012, making gonorrhea the sixth most common infectious disease [4] and the first most common STD [5].

Management of *N. gonorrhoeae* infection is a serious challenge in the setting of increasing antimicrobial resistance (AMR) [6–8]. The importance of AMR surveillance has been extensively elucidated [9, 10], particularly as treatment options for gonorrhea decreased because of the spread of resistance to sulfonamides, penicillin, tetracycline, and quinolone.

China established a national surveillance program to monitor AMR in 1987. Twenty-five *N. gonorrhoeae* AMR surveillance sentinel sites were distributed in 25 provinces of China based on the medical resource distribution and geographical representativeness, which were established by the Chinese Center for Disease Control and Prevention (CDC). *N. gonorrhoeae* resistance cases were sent to Chinese CDC by sentinel sites. Since 1992, China has been a part of WHO Western Pacific Regional (WPR) Gonococcal Antimicrobial Surveillance Programme. However, we found that only two studies reported *N. gonorrhoeae* resistance to antimicrobials based on nationwide data from the surveillance program [11, 12]. Whereas, more than a hundred reports on *N. gonorrhoeae* AMR were based on data from one or a few surveillance sentinel sites, and these studies had obvious differences in settings, methods, findings, and some other characteristics. We conducted this systematic review in order to provide a comprehensive understanding of *N. gonorrhoeae* resistance to antmicrobials in China.

## Methods

This meta-analysis was conducted according with the checklist of the Meta-Analysis of Observational Studies in Epidemiology Guideline [13].

### Search strategy

Two independent researchers (Y.W.C and T.T.Y) searched four Chinese biomedical databases (China Biology Medicine disc [CBMdisc] (1978–), China National Knowledge Infrastructure [CNKI] (1915–), VIP Information/Chinese Scientific Journals database (1989–) and Wanfang database (1998–)) and PubMed (1966–) to identify relevant studies from their respectively inception to November 7, 2014. Search terms (in Chinese) included “*Neisseria gonorrhoeae*” or “gonorrhea”, and their combinations with “resistant” or “resistance” or “susceptible” or “susceptibility” in Chinese databases. We used the string ‘(*Neisseria gonorrhoeae* or gonococc* or gonorrh*) AND (resistan* or sensitiv* or susceptib*) AND (China or Chinese)’ in PubMed. In addition, relevant references from each study were retrieved and further added to the analysis.

### Inclusion criteria

The included studies met the following criteria: (1) an original study published in Chinese or English; (2) conducted in mainland China; (3) determined minimum inhibitory concentrations (MICs) by using the agar dilution method; (4) specified the total number of tested *N. gonorrhoeae* isolates; (5) reported the AMR rate in *N. gonorrhoeae* isolates, or implied it by indicating their MICs, following the criteria by WHO WPR Resistance Surveillance Programme guidelines[14] or Clinical and Laboratory Standards Institute standards; (6) tested more than 30 *N. gonorrhoeae* isolates in order to guarantee the quality of testing; and (7) isolated *N. gonorrhoeae* strains from adults (age ≥18 years).

### Data extraction

The following data was extracted from each study: name of first author, publication year, study region, number of tested isolates, and resistance rate. Data from the included studies were independently extracted by two authors (X.Y.S and J.L) and any discrepancies were resolved through the introduction of a third reviewer (X.X.Y).

### Quality assessment

The methodological quality of the included studies was assessed with the recommended approach of the WHO [15]. Briefly, we assessed (1) whether the study described the method of identifying *N. gonorrhoeae* isolates;(2) whether the study specified the location where *N. gonorrhoeae* isolates were collected;(3) whether the study specified the collection period of the isolates; (4) whether the study described the population from which *N. gonorrhoeae* isolates were obtained;(5) whether the study included at least 100 tested *N. gonorrhoeae* isolates;(6) whether the study utilized control strains recommended by WHO in determining MICs with agar dilution method.

The quality assessment scores of the included studies are shown in Additional file 1: Table S1. Each criterion was rated 1 point if a study satisfied the WHO’s recommendation. Two independent reviewers assessed the quality of the included studies. We considered studies that scored 5 or higher as “high quality”, 3 or 4 as “moderate quality”, and 2 or lower as ‘low quality’.

### Statistical analysis

*N. gonorrhoeae* resistance rates to antimicrobials with their corresponding 95 % confidence intervals (CIs) were calculated using the random-effects model. Where a study reported results for years separately, these were regarded as separate reports. Subgroup analyses were carried out by year. If the number of reports in 1 year was less than 3, these reports were subsumed in the next year. For example, only one report about *N. gonorrhoeae* resistance to tetracycline in 2002; thus, we subsumed this report in 2003 to conduct a meta-analysis. In our study, considering that reports published before 1995 and after 2012 were scarce and the number of reports was less than 3 in any year before 1995 and after 2012, we have subsumed the reports published before 1995 and after 2012 in one subgroup to make our results more understandable.

Statistical heterogeneity among studies was evaluated by using the *I*^*2*^ statistic, where values of 25, 50 and 75 % represent cut-off points for low, moderate and high degrees of heterogeneity, respectively [16]. Publication bias was assessed by using the Begg test [17] and the Egger test [18]. All statistical analyses were performed by using Comprehensive Meta Analysis V3. All tests were two-sided with a significance level of 0.05.

## Results

### Literature search

**Fig. 1:**
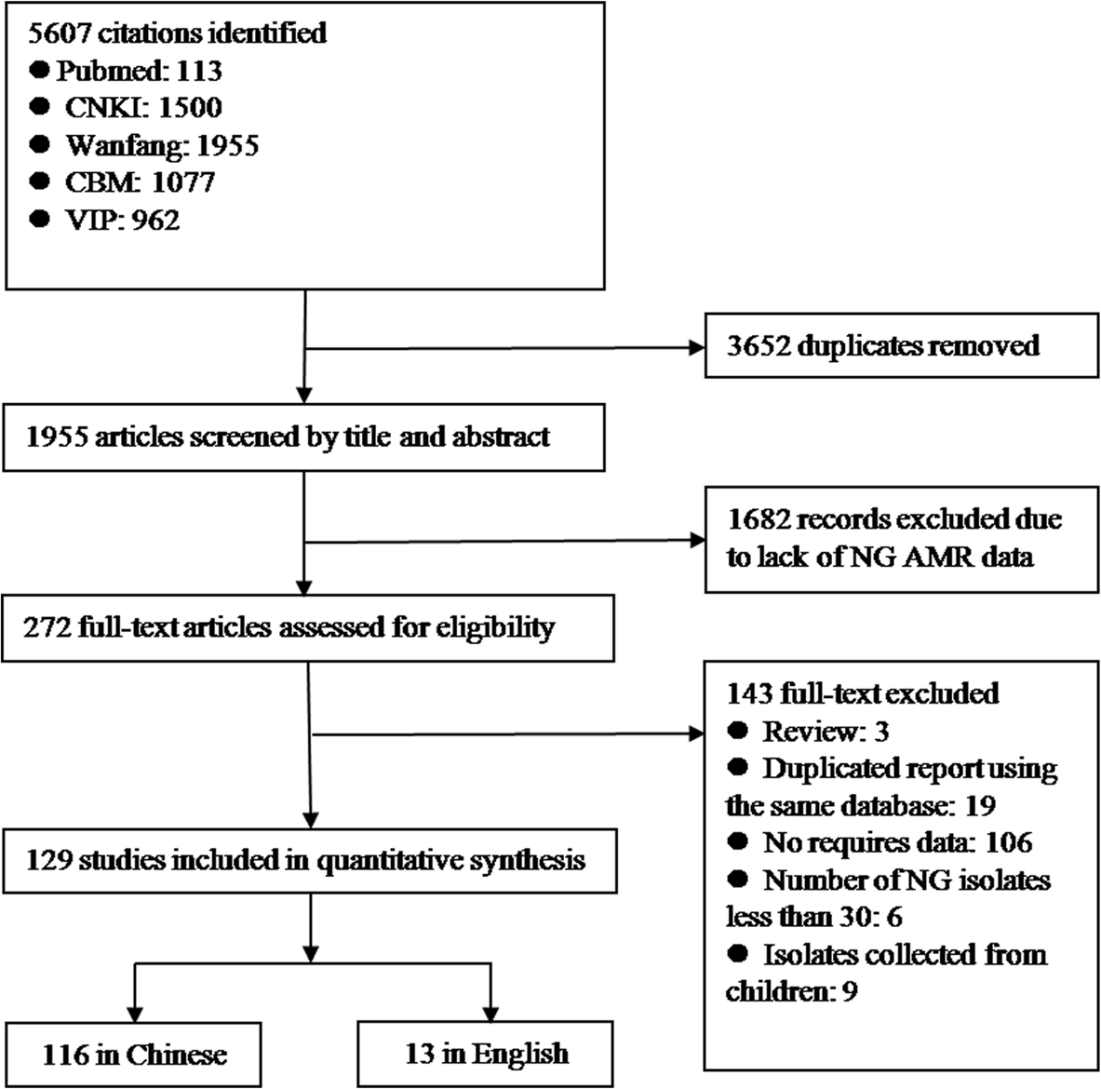
Process of selecting published studies for the meta-analysis

We present the study selection process in Fig. 1. The search identified 5,607 unique articles from PubMed, CNKI, CBMdisc, VIP, and WanFang, of which 270 were identified as potentially relevant. After retrieving the full text for further evaluation, 127 studies that reported *N. gonorrhoeae* AMR rates were included.

### Study characteristics

The main study characteristics and quality assessment results are shown in Additional file 1: Table S1. Of the127 studies (Additional file 2: Table S2), 12 were in English and 115 were in Chinese. These studies were published between 1991 and 2014. In these studies, 42,509 *N. gonorrhoeae* isolates were examined for their resistance to one or more antimicrobials used for the treatment of gonorrhea. Of the 127 studies, 90 were deemed high-quality studies; 34, moderate-quality studies; and 3, low-quality studies. The *N. gonorrhoeae* resistance rates to penicillin, tetracycline, ciprofloxacin, ceftriaxone and spectinomycin were estimated in our study.

### *N. gonorrhoeae* resistance to antimicrobials

**Fig. 2:**
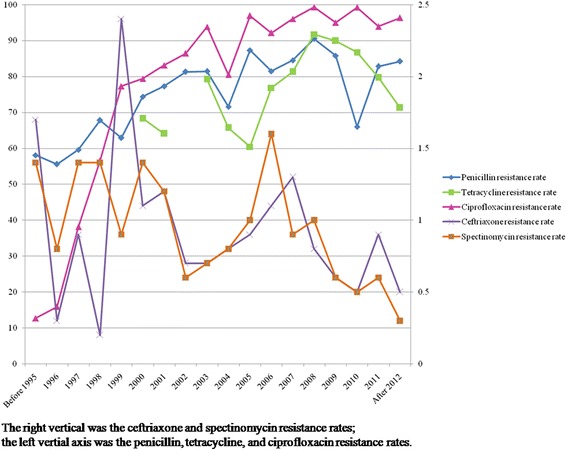
*N. gonorrhoeae* resistance to penicillin, tetracycline, ciprofloxacin, ceftriaxone and spectinomycin, 1995–2012

In 91 studies, 154 reports were found on *N. gonorrhoeae* resistance to penicillin. The pooled *N. gonorrhoeae* resistance rate to penicillin was 58.1 % (95 % CI: 50.3–65.8 %) before 1995 and increased up to 84.2 % (95 % CI: 79.7–88.8 %) in 2012 (Fig. 2).

According to 73 reports, the *N. gonorrhoeae* resistance rate to tetracycline fluctuated over time. The resistance rate was 68.3 % (95 % CI: 58.7–78.0 %) before 2003 and picked in 2008 (91.7 %, 95 % CI: 86.4–97.0 %; Fig. 2). Between 1994 and 2013, 221 reports from 113 studies were about *N. gonorrhoeae* resistance to ciprofloxacin. The *N. gonorrhoeae* resistance rate to ciprofloxacin reached 93.8 % (95 % CI: 91.9–95.7 %) in 2003 and fluctuated between 92.1 % (95 % CI: 90.1–94.0 %) and 99.3 % (95 % CI: 98.9–99.7 %) between 2004 and 2012 (Fig. 2).

In 103 studies, 205 reports estimated *N. gonorrhoeae* resistance to ceftriaxone. The pooled resistance rate was 1.7 % (95 % CI: 0.5–5.7 %) before 1995 and 0.5 % (95 % CI: 0.2–1.4 %) in 2012 (Fig. 2). *N. gonorrhoeae* resistance to spectinomycin was reported in 113 studies, with 33,485 *N. gonorrhoeae* isolates. The pooled *N. gonorrhoeae* resistance rate to spectinomycin was 0.3 % (95 % CI: 0.2–0.8 %; Fig. 2). Data on *N. gonorrhoeae* resistance to antimicrobials are shown in Additional file 3: Table S3.

### Publication bias

Evidence of high heterogeneity was observed for each antimicrobial agent in our study (*I*^*2*^ ranged from 64.89 to 95.80 %, *P* = 0.001). The Begg rank correlation test and Egger linear regression test indicated evidence of publication bias among studies for each antimicrobial agent (*P* < 0.05 for both tests); Additional file 4: Table S4).

## Discussion

Based on data from 127 studies, including 42,509 tested *N. gonorrhoeae* isolates, *N. gonorrhoeae* resistance to the previously recommended first-line antimicrobials penicillin, tetracycline and ciprofloxacin reached as high as 84.2, 71.3, and 96.3 %, respectively, in China. The *N. gonorrhoeae* resistance rates to penicillin and tetracycline were higher in China than in other Asian countries such as Vietnam [9], India, Pakistan and Bhutan [19] during the same period. Ciprofloxacin-resistant *N. gonorrhoeae* isolates in China were much higher than those reported in Laos [20–22] and Vietnam [20–26], and were comparable with those in Thailand [9, 27, 28]. *N. gonorrhoeae* resistance rate to spectinomycin remains very low, which is in accordance with WHO report [29] and previous studies from other countries in the Western Pacific Region [30–33].

The WHO recommends that routine use of an antimicrobial for treatment should be discontinued when therapeutic failure and/or AMR rate reaches a level of 5 % [34]. The unremitting development of resistance to multiple antimicrobials in *N. gonorrhoeae* isolates has motivated ongoing modification of treatment guidelines in China. Penicillin was recommended as the first choice of treatment of *N. gonorrhoeae* infections in 1989. By 1995, the *N. gonorrhoeae* resistance rate to penicillin was nearly 60 %. The recommended treatment of gonorrhea was then changed to ciprofloxacin in 1995. Afterwards, the *N. gonorrhoeae* resistance rate to ciprofloxacin increased sharply and reached 79.4 % by 2000. Many studies suggest that the widespread use of antimicrobials was an important cause in the development of AMR [35–38]. We hypothesize that *N. gonorrhoeae* AMR might be contributed to the widespread use of penicillin and ciprofloxacin after they have been recommended for clinical use. In response to extensive *N. gonorrhoeae* resistance to penicillin and ciprofloxacin, ceftriaxone and spectinomycin were recommended as the first-line antimicrobials for the treatment of gonorrhea in 2000 in China. Fortunately, according to our data, *N. gonorrhoeae* resistance to both ceftriaxone and spectinomycin is not widespread. The *N. gonorrhoeae* resistance rates to ceftriaxone and spectinomycin were 0.5 and 0.3 %, respectively, in 2012.

The main reason for the rapid increase of *N. gonorrhoeae* resistance rates to penicillin and ciprofloxacin after they have been recommended for clinical use is likely their indiscriminate use. Gonorrhea was considered to be caused by the sexual misconduct in China in the 1990s; and because of stigma, patients trended to administer antimicrobial treatment by themselves or visited non-formal medical institutions such as unregistered private clinics. Moreover, some patients did not complete the full treatment course, increasing the chance for AMR to develop. In addition, at the time, antimicrobial management policies and effective supervision over physicians’ prescribing behaviors were lacking in China. For example, patients could buy antimicrobials in drug stores without a prescription. Indeed, an association between self-prescribed antimicrobial use and gonococcal AMR was observed in Philippines [39].

Recognizing the rapid spread and serious consequences of AMR [40], the Ministry of Health of China promulgated “The Guidelines on the Clinical Application of Antimicrobials” in 2004 and “The Guidelines on Prescription Management” in 2006. The Ministry also issued “Prescription Management and Evaluation Standards in Clinical Practice” in 2010. For STDs, the Chinese CDC formulated “The Guidelines for Diagnosis and Treatment of Sexually Transmitted Disease” in 2006; the Ministry of Health modified and promulgated “Prevention and Management of Sexually Transmitted Disease” in 2012, which defined legal responsibility for relevant departments and personnel, and explicitly stated that medical institutions should further standardize the treatment of gonorrhea. In addition, the government strengthened health education on the importance of timely and standardized treatment of gonorrhea. These measures may have potentially contributed to the low resistance rates to ceftriaxone and spectinomycin. However, given that treatment failures with ceftriaxone have been reported not only in China [41–43] but also in Japan, France, and Spain [44–46], and spectinomycin-resistant *N. gonorrhoeae* strains have been detected in China [47–51] and other countries [52, 26], clinicians should closely monitor for the resistance status of patients treated with ceftriaxone and spectinomycin.

Our study has a number of limitations: (1) the included studies were conducted in various regions, mostly with variable sample sizes, which introduced substantial heterogeneity to the data obtained; (2) all tested *N. gonorrhoeae* isolates were collected from patients who attended STD clinics or hospital outpatient departments, which may introduce sample bias for the patients may have a higher prevalence of *N. gonorrhoeae* AMR than the general population; and (3) demographic information such as sex and age and information regarding “risk patient groups” or “risk factors” were lacking.

## Conclusions

*N. gonorrhoeae* resistance rates to penicillin, tetracycline and ciprofloxacin were high in China, while the resistance rates to ceftriaxone and spectinomycin remained lower than 5 %. Therefore, ceftriaxone and spectinomycin can be recommended as effective therapy for gonorrhea in China. Nevertheless, considering the emergency of ceftrixone and spectinomycin resistant strains in various regions, it is essential to strengthen *N. gonorrhoeae* resistance surveillance and update treatment guidelines timely based on new evidence as antimicrobial resistance develops.

### Ethics approval and consent to particapate

Not applicable.

### Consent for publication

Not applicable.

### Availability of data materials

The datasets supporting the conclusions of this article are included within the article and its additional files.

## Additional files

Additional file 1: Table S1. Characteristics and quality assessment of studies included in the systematic review. (DOCX 48 kb) Additional file 2: Table S2. The included studies. (DOCX 23 kb) Additional file 3: Table S3. Antimicrobial resistance rate of Neisseria Gonorrhoeae isolates. (DOCX 24 kb) Additional file 4: Table S4. The Begg and Egger test of heterogeneity. (DOCX 14 kb)

## Abbreviations

AMR: antimicrobial resistance
CBMdisc: China Biology Medicine disc
CI: confidence interval
CNKI: China National Knowledge Infrastructure
*Neisseria gonorrhoeae*: *N. gonorrhoeae*
STD: sexually transmitted disease
WHO: World Health Organization
