# Supplementary material for: Antimicrobial resistance in Neisseria gonorrhoeae in China: a meta-analysis
Source: BMC Infect Dis. 2016 Mar 3;16:108. doi: 10.1186/s12879-016-1435-0 (PMC4778342; doi:10.1186/s12879-016-1435-0)
Supplement: Additional file 1: Table S1. — Characteristics and quality assessment of studies included in the systematic review. (DOCX 48 kb) [file 12879_2016_1435_MOESM1_ESM.docx]

**Table S1 Characteristics and quality assessment of studies included in the systematic review**

| Study source | Isolate collection period | Location | Population^a^ | No. of isolates | Drug | Quality assessment | | | | | | |
| --- | --- | --- | --- | --- | --- | --- | --- | --- | --- | --- | --- | --- |
|  |  |  |  |  |  | Isolates identification | Location | Isolates collection period | Population | No. of NG isolates≥100 | Control strains | Total scores |
| Bangyong Zhu et al, 2014 | 2000-2012 | Guangxi | Patients | 923 | Cip, Cef, Spe | 1 | 1 | 1 | 0 | 1 | 1 | 5 |
| Heping Zheng et al, 2014 | 2002-2011 | Guangdong | Patients | 1257 | Pen, Cip, Cef, Spe, | 1 | 1 | 1 | 0 | 1 | 1 | 5 |
| Haihua Huang et al, 2014 | 2010-2012 | Guangdong | Patients | 127 | Pen,Tet, Cip, Cef, Spe | 1 | 1 | 0 | 0 | 1 | 1 | 4 |
| Xiaohua Wu et al, 2014 | 2011-2013 | Guangdong | Patients | 493 | Pen,Tet, Cip, Cef, Spe | 1 | 1 | 1 | 0 | 1 | 1 | 5 |
| Yanwa Li et al, 2014 | 2007 | Guangdong | Patients | 98 | Pen, Tet | 0 | 1 | 1 | 0 | 0 | 0 | 2 |
| Xiaofeng Liu et al, 2014 | 2011-2013 | Guangdong | Patients | 192 | Pen,Tet, Cip, Cef, Spe | 1 | 1 | 1 | 0 | 1 | 1 | 5 |
| Wenling Cao et al, 2014 | 2012/12-2013/11 | Guangdong | Patients | 100 | Pen, Cip, Cef, Spe, | 1 | 1 | 1 | 0 | 1 | 1 | 5 |
| Wenwei Wu et al, 2014 | 2013 | Guangdong | Patients | 85 | Pen,Tet, Cip, Cef, Spe | 1 | 1 | 0 | 0 | 0 | 1 | 3 |
| Xinghai Zhao et al, 2014 | 2009-2013 | Hebei | Women | 118 | Pen,Tet, Cip, Cef, Spe | 1 | 1 | 1 | 1 | 1 | 1 | 6 |
| Chixing Guo et al, 2014 | 2008/1-2012/12 | Guangdong | Patients | 436 | Pen,Tet, Cip, Cef, Spe | 1 | 1 | 1 | 0 | 1 | 1 | 5 |
| Na Zhong et al, 2014 | 2011/1-2012/10 | Hainan | Patients | 214 | Cip, Cef, Spe | 1 | 1 | 1 | 0 | 1 | 1 | 5 |
| Ying Jiang et al, 2013 | 2008-2012 | Guangdong | Patients | 103 | Cip, Cef, Spe | 1 | 1 | 1 | 0 | 1 | 1 | 5 |
| Wenfeng Chen et al, 2013 | 2012 | Guangdong | Mix | 204 | Pen,Tet, Cip, Cef, Spe | 1 | 1 | 1 | 1 | 1 | 1 | 6 |
| Na Zhong et al, 2013 | 2006-2011 | Hainan | Patients | 384 | Cip, Cef, Spe | 1 | 1 | 1 | 0 | 1 | 1 | 5 |
| Xiaowei Zhou et al, 2013 | 2009-2011 | Liaoning | Patients | 320 | Cip, Cef, Spe | 1 | 1 | 1 | 0 | 1 | 1 | 5 |
| Xingzhong Wu et al, 2013 | 2011-2012 | Guangdong | Patients | 525 | Pen,Tet, Cip, Cef, Spe | 1 | 1 | 1 | 0 | 1 | 1 | 5 |
| Baozhu Yu et al, 2013 | 2012 | Guangdong | Patients | 137 | Pen,Tet, Cip, Cef, Spe | 1 | 1 | 1 | 0 | 1 | 1 | 5 |
| Yuhua Gong et al, 2013 | 2011-2012 | Guangdong | Patients | 118 | Pen,Tet, Cip, Cef, Spe | 1 | 1 | 1 | 0 | 1 | 0 | 4 |
| Lihao Li et al, 2013 | 2010/1-2013/1 | Guangdong | Patients | 212 | Pen,Tet, Cip, Cef, Spe | 0 | 1 | 1 | 0 | 1 | 0 | 3 |
| Lijun Zhang et al, 2013 | 2008-2011 | Guangdong | Patients | 732 | Cip, Cef, Spe | 1 | 1 | 1 | 0 | 1 | 1 | 5 |
| Xiaodong Li et al, 2012 | 2009/12-2010/12 | Guangdong | Patients | 83 | Cip, Cef, Spe | 1 | 1 | 1 | 0 | 0 | 1 | 4 |
| Aiming Wu et al, 2012 | 2010 | Guangdong | Mix | 200 | Pen,Tet, Cip, Cef, Spe | 1 | 1 | 1 | 1 | 1 | 1 | 6 |
| Li'e Lao et al, 2012 | 2010-2011 | Guangdong | Patients | 150 | Pen,Tet, Cip, Cef, Spe | 1 | 1 | 1 | 0 | 1 | 1 | 5 |
| Xiaohong Su et al, 2007 | 1999-2006 | Jiangsu | Patients | 1108 | Pen,Cip, Cef, Spe | 1 | 1 | 1 | 0 | 1 | 1 | 5 |
| Xingzhong Wu et al, 2012 | 2011 | Guangdong | Patients | 429 | Pen,Tet, Cip, Cef, Spe | 1 | 1 | 1 | 0 | 1 | 1 | 5 |
| Guoming Li et al, 2000 | 1998-1999 | Guangdong | Mix | 98 | Pen,Tet, Cip, Cef, Spe | 1 | 1 | 1 | 1 | 0 | 1 | 5 |
| Wenling Cao et al, 2000 | 1997/9-1998/8 | Guangdong | Men | 203 | Pen, Cip, Cef, Spe | 1 | 1 | 1 | 1 | 1 | 1 | 6 |
| Shunzhang Ye et al, 2001 | 1993-1998 | Mix | Patients | 3186 | Pen,Tet, Cip, Cef | 1 | 1 | 1 | 0 | 1 | 1 | 5 |
| Shunzhang Ye et al, 1994 | 1987-1992 | Mix | Patients | 1327 | Pen | 1 | 1 | 1 | 0 | 1 | 1 | 5 |
| Heping Zheng et al, 2003 | 1996-2001 | Guangdong | Patients | 793 | Pen, Cip, Cef, Spe | 1 | 1 | 1 | 0 | 1 | 1 | 5 |
| Tiejun Zhang et al, 2007 | 2005/4-2006/4 | Shanghai | Patients | 80 | Cip | 1 | 1 | 1 | 0 | 0 | 1 | 4 |
| Bei Wang et al, 2006 | 2003/5-2003/12 | Jiangsu | Patients | 95 | Pen,Tet, Cip, Cef, Spe | 1 | 1 | 1 | 0 | 0 | 0 | 3 |
| Yang Yang et al, 2006 | 2004/11-2005/5 | Shanghai | Men | 159 | Pen,Tet, Cip, Cef, Spe | 1 | 1 | 1 | 1 | 1 | 1 | 6 |
| Shaochun Chen et al, 2014 | 2007-2008 | Jiangsu | Patients | 278 | Cip,Spe | 1 | 1 | 1 | 0 | 1 | 1 | 5 |
| Haili Xie et al, 2012 | 2009-2011 | Guangxi | Mix | 96 | Cip, Cef, Spe | 1 | 1 | 1 | 1 | 0 | 1 | 5 |
| Chengguang Zhang et al, 2011 | 2008/1-2010/1 | Jiangsu | Mix | 60 | Pen,Cip, Cef, Spe | 1 | 1 | 1 | 1 | 0 | 1 | 5 |
| Xiaofeng Liu et al, 2011 | 2010/1-2011/11 | Guangdong | Patients | 105 | Pen,Tet, Cip, Cef, Spe | 1 | 1 | 1 | 0 | 1 | 1 | 5 |
| Yufeng Niu et al, 2011 | 2009/1-2009/12 | Jiangsu | Patients | 120 | Pen, Cip, Cef, Spe | 1 | 1 | 1 | 0 | 1 | 1 | 5 |
| Jian Xie et al, 2011 | 2006/1-2010/10 | Guangxi | Patients | 233 | Tet, Cip, Cef | 1 | 1 | 1 | 0 | 1 | 1 | 5 |
| Yan Han et al, 2011 | 2008 | Mix | Mix | 951 | Cip, Cef, Spe | 1 | 1 | 1 | 1 | 1 | 1 | 6 |
| Zongxia Yu et al, 2011 | 2008/2-2009/3 | Jiangsu | Patients | 105 | Pen,Tet, Cip, Cef, Spe | 1 | 1 | 1 | 0 | 1 | 1 | 5 |
| Fenglian Feng et al, 2011 | 2002-2009 | Shanxi | Mix | 647 | Cip, Cef, Spe | 1 | 1 | 1 | 1 | 1 | 1 | 6 |
| Wenling Cao et al, 2011 | 2008/1-2009/12 | Guangdong | Patients | 88 | Pen, Cip, Cef, Spe | 1 | 1 | 1 | 0 | 0 | 1 | 4 |
| Wenwei Wu et al, 2010 | 2005/1-2009/1 | Guangdong | Patients | 128 | Pen,Tet, Cip, Cef, Spe | 0 | 1 | 1 | 0 | 1 | 1 | 4 |
| Feng Wu et al, 2010 | 2005/1-2008/1 | Shandong | Mix | 135 | Pen,Tet, Cef, Spe | 1 | 1 | 1 | 1 | 1 | 0 | 5 |
| Junming Pei et al, 2010 | 2009 | Guangdong | Patients | 92 | Pen,Tet, Cip, Cef, Spe | 1 | 1 | 1 | 0 | 0 | 0 | 3 |
| Ruixue Chu et al, 2010 | 2009/1-2009/12 | Shandong | Patients | 52 | Tet, Cip, Cef, Spe | 1 | 1 | 1 | 0 | 0 | 1 | 4 |
| Donghong Tan et al, 2010 | 2000/1-2008/12 | Guangxi | Mix | 771 | Cip, Cef, | 1 | 1 | 1 | 1 | 1 | 1 | 6 |
| Shunxin Cai et al, 2010 | 2009 | Guangdong | Mix | 200 | Pen,Tet, Cip, Cef, Spe | 1 | 1 | 1 | 1 | 1 | 1 | 6 |
| Lijun Zhang et al, 2010 | 2008/7-2008/10 | Guangdong | Patients | 196 | Cip, Cef, Spe | 1 | 1 | 1 | 0 | 1 | 1 | 5 |
| Jianmei Zhao et al, 2009 | 2006/6-2007/8 | Zhejiang | Mix | 118 | Cip, Cef, Spe | 1 | 1 | 1 | 1 | 1 | 0 | 5 |
| Xiuqin Dai et al, 2009 | 2006 | Mix | Patients | 192 | Spe | 1 | 1 | 1 | 0 | 1 | 1 | 5 |
| Shiping Lin et al, 2009 | 2006/1-2007/6 | Guangdong | Patients | 240 | Pen,Tet, Cip, Cef, Spe | 1 | 1 | 1 | 0 | 1 | 0 | 4 |
| Wenling Cao et al, 2009 | 2007/1-2008/12 | Guangdong | Patients | 74 | Pen, Cef, Spe | 1 | 1 | 1 | 0 | 0 | 1 | 4 |
| Yanwa Li et al, 2009 | 2007/1-2007/11 | Guangdong | Patients | 98 | Pen,Tet, Cip, Cef, Spe | 1 | 1 | 1 | 0 | 0 | 1 | 4 |
| Xin Zhu et al, 2009 | 2008/1-2008/12 | Guangdong | Mix | 80 | Pen,Tet, Cip, Cef, Spe | 1 | 1 | 1 | 1 | 0 | 1 | 5 |
| Xiuqin Dai et al, 2009 | 2006/12-2007/11 | Jiangsu | Mix | 202 | Cip, Cef, Spe | 1 | 1 | 1 | 1 | 1 | 1 | 6 |
| Jieru Pan et al, 2009 | 2007-2008 | Guangdong | Patients | 108 | Pen,Tet, Cip, Cef, Spe | 1 | 1 | 1 | 0 | 1 | 1 | 5 |
| Chixing Guo et al, 2009 | 2008 | Guangdong | Patients | 107 | Pen,Tet, Cip, Cef, Spe | 1 | 1 | 1 | 0 | 1 | 1 | 5 |
| Kehua Xu et al, 2009 | 2006/1-2008/7 | Jiangxi | Patients | 133 | Pen,Tet, Cip, Cef, Spe | 1 | 1 | 1 | 0 | 1 | 1 | 5 |
| Jian Xie et al, 2009 | 1996-2008 | Guangxi | Patients | 970 | Cip | 1 | 1 | 1 | 0 | 0 | 1 | 4 |
| Cunjun Hou et al, 2008 | 2005/1- 2006/11 | Shandong | Patients | 148 | Cip, Cef, Spe | 1 | 1 | 1 | 0 | 1 | 1 | 5 |
| Xiuqin Wang, et al, 2008 | 2005/1-2007/3 | Shandong | Patients | 165 | Tet, Cip, Cef, Spe | 1 | 1 | 1 | 0 | 1 | 1 | 5 |
| Xiaorong Ren et al, 2008 | 2006/2-2006/10 | Guangdong | Patients | 98 | Pen, Cip, Cef, Spe | 1 | 1 | 1 | 0 | 0 | 1 | 4 |
| Yiting Ma et al, 2008 | 2005/4-2006/4 | Guangdong | Patients | 113 | Pen, Cip, Cef | 1 | 1 | 1 | 0 | 1 | 1 | 5 |
| Xingzhong Wu et al, 2008 | 2007 | Guangdong | Patients | 123 | Pen,Tet, Cip, Cef, Spe | 1 | 1 | 1 | 0 | 1 | 1 | 5 |
| Dan Ke et al, 2008 | 2006/1-2006/12 | Chongqing | Mix | 93 | Pen,Tet, Cip, Cef, Spe | 1 | 1 | 1 | 1 | 0 | 0 | 4 |
| Na Zhong et al, 2008 | 2004/11-2007/6 | Hainan | Patients | 129 | Cip, Cef, Spe | 1 | 1 | 1 | 0 | 1 | 1 | 5 |
| Kun Pan et al, 2008 | 2008/1-2008/5 | Shandong | Mix | 100 | Pen,Tet, Cip, Cef | 1 | 1 | 1 | 1 | 1 | 1 | 6 |
| Gufen Fang et al, 2007 | 2001/1-2006/12 | Hunan | Mix | 266 | Pen,Tet, Cip, Cef, Spe | 1 | 1 | 1 | 1 | 1 | 1 | 6 |
| Jianmei Zhao et al, 2007 | 2005/6-2006/5 | Zhejiang | Mix | 78 | Pen,Tet, Cip, Cef, Spe | 1 | 1 | 1 | 1 | 0 | 1 | 5 |
| Bin Wu et al, 2007 | 2003/12-2005/6 | Neimenggu | Mix | 86 | Pen, Cip, Cef, Spe | 1 | 1 | 1 | 1 | 0 | 1 | 5 |
| Xingzhong Wu et al,2007 | 2005 | Guangdong | Mix | 104 | Pen,Tet, Cip, Cef, Spe | 1 | 1 | 0 | 1 | 1 | 1 | 5 |
| Xiaorong Ren et al, 2007 | 2006/2-2006/10 | Shanxi | Mix | 102 | Pen, Cip, Cef, Spe | 1 | 1 | 1 | 1 | 1 | 1 | 6 |
| Zhensheng Wang et al, 2007 | 2001-2006 | Fujian | Women | 1206 | Cip, Cef, Spe | 0 | 1 | 1 | 1 | 1 | 1 | 5 |
| Yating Tu et al, 2006 | 2004/4-2004/12 | Hubei | Patients | 70 | Pen,Tet, Cip, Cef, Spe | 1 | 1 | 1 | 0 | 0 | 1 | 4 |
| Wenling Cao et al, 2006 | 2005 | Guangdong | Patients | 110 | Pen, Cip, Cef, Spe | 1 | 1 | 1 | 0 | 1 | 1 | 5 |
| Xincheng Wang et al, 2006 | 2004/2-2006/3 | Jilin | Mix | 153 | Pen,Tet, Cef, Spe, | 0 | 1 | 1 | 1 | 1 | 1 | 5 |
| Wenming Zhou et al, 2006 | 2002/1-2003/3 | Anhui | Mix | 87 | Cip | 0 | 1 | 1 | 1 | 1 | 1 | 5 |
| Gang Yong et al, 2006 | 2000-2004 | Sichuan | Patients | 978 | Pen, Cip, Cef, Spe | 1 | 1 | 1 | 0 | 1 | 1 | 5 |
| Xingzhong Wu et al, 2006 | 2004-2005 | Guangdong | Patients | 527 | Pen,Tet, Cip, Cef, Spe | 1 | 1 | 1 | 0 | 1 | 1 | 5 |
| Hui Gao et al, 2006 | 1994 2004 | Hebei | Mix | 302 | Pen,Tet, Cip, Cef, Spe | 1 | 1 | 1 | 1 | 1 | 0 | 5 |
| Fenglian Feng et al, 2005 | 2002/1/12 | Shanxi | Mix | 105 | Cip, Cef, Spe | 0 | 1 | 1 | 1 | 1 | 1 | 5 |
| Zizhong Xiong et al, 2005 | 2003、10-12 | Anhui | Patients | 35 | Pen,Tet, Cip, Cef, Spe | 0 | 1 | 1 | 0 | 0 | 0 | 2 |
| Yongjun Yang et al, 2005 | 2001/1-2003/8 | Shandong | Mix | 368 | Pen,Cip, Cef, Spe | 1 | 1 | 1 | 1 | 1 | 1 | 6 |
| Shenghui Yang et al, 2005 | 2003/1-2004/8 | Hunan | Patients | 101 | Pen,Tet, Cip, Cef, Spe | 1 | 1 | 1 | 0 | 1 | 1 | 5 |
| Xibao Zhang et al, 2005 | 2003/1-2003/12 | Guangdong | Patients | 117 | Cip, Cef, | 1 | 1 | 1 | 0 | 1 | 1 | 5 |
| Wenling Cao et al, 2005 | 2003/12-2004/11 | Guangdong | Patients | 127 | Pen, Cip, Cef, Spe | 1 | 1 | 1 | 0 | 1 | 1 | 5 |
| Yang Yang et al, 2005 | 2001-2003 | Shanghai | Patients | 742 | Pen, Cip, Cef, Spe | 1 | 1 | 1 | 0 | 1 | 1 | 5 |
| Qin He et al, 2005 | 2000/11-2004/12 | Guangdong | Mix | 246 | Pen, Cip, Cef | 1 | 1 | 1 | 1 | 1 | 1 | 6 |
| Fan Li et al, 2004 | 2000/1-2002/12 | Jiangxi | Patients | 120 | Pen,Tet, Cip, Cef, Spe | 1 | 1 | 1 | 0 | 1 | 1 | 5 |
| Xingzhong Wu et al, 2004 | 2002-2003 | Guangdong | Patients | 107 | Pen,Tet, Cip, Cef, Spe | 1 | 1 | 0 | 0 | 1 | 1 | 4 |
| Wenling Cao et al, 2004 | 2002/12-2003/11 | Guangdong | Patients | 116 | Cip, Cef, Spe | 1 | 1 | 1 | 0 | 1 | 1 | 5 |
| Xingzhong Wu et al, 2004 | 2000-2003 | Guangdong | Mix | 396 | Cip | 1 | 1 | 0 | 1 | 1 | 1 | 5 |
| Shenghui Yang et al, 2004 | 1999/1-2000/8 | Guangdong | Patients | 95 | Cef, Spe | 1 | 1 | 1 | 0 | 0 | 1 | 4 |
| Wei Wang et al, 2004 | 1999-2000 2002-2003 | Neimenggu | Mix | 122 | Pen, Cip, Cef, Spe | 1 | 1 | 0 | 1 | 0 | 0 | 3 |
| Xiaohong Su et al, 2004 | 1999-2002 | Jiangsu | Mix | 417 | Cip, Cef, Spe | 0 | 1 | 0 | 1 | 1 | 1 | 4 |
| Wenling Cao et al, 2004 | 1998/12-2003/11 | Guangdong | Mix | 707 | Pen, Cip, Cef, Spe | 1 | 1 | 1 | 1 | 1 | 1 | 6 |
| Yatian Qin et al, 1991 | 1989/11-1990/7 | Chongqing | Mix | 38 | Pen, Spe | 1 | 1 | 1 | 1 | 0 | 0 | 4 |
| Kezhou Gu et al, 1992 | 1989/5-1990/10 | Zhejiang | Patients | 81 | Pen, Spe | 1 | 1 | 1 | 0 | 0 | 1 | 4 |
| Xiaohong Su et al, 1996 | 1994/7-1995/6 | Mix | Patients | 390 | Pen,Tet, Cip, Cef, Spe | 1 | 1 | 1 | 0 | 1 | 1 | 5 |
| Shunzhang Ye et al, 1997 | 1989-1996 | Mix | Patients | 1631 | Spe | 1 | 1 | 1 | 0 | 1 | 1 | 5 |
| Xiaohong Su et al, 1997 | 1994/7-1996/7 | Mix | Patients | 535 | Cip | 0 | 1 | 1 | 0 | 1 | 1 | 4 |
| Qingfang Xu et al, 1997 | 1995/1-1995/2 1994/12-2995/3 | Shanghai | Patients | 117 | Pen,Tet, Cef, Spe | 0 | 1 | 1 | 0 | 1 | 1 | 4 |
| Ding'an Xu et al, 1997 | 1995/6-1996/6 | Jiangsu | Patients | 163 | Pen,Tet, Cip, Cef, Spe | 1 | 1 | 1 | 0 | 1 | 1 | 5 |
| Zhiqin Gao et al, 1997 | 1996/10-1996/12 | Shanghai | Patients | 206 | Pen,Tet, Cip, Cef, Spe | 1 | 1 | 1 | 0 | 1 | 1 | 5 |
| Heping Zheng et al, 1998 | 1996 | Guangdong | Patients | 201 | Pen,Tet, Cip, Cef, Spe | 1 | 1 | 1 | 0 | 1 | 1 | 5 |
| Heping Zheng et al, 1998 | 1988-1991 1996-1997 | Guangdong | Patients | 439 | Pen | 1 | 1 | 1 | 0 | 1 | 1 | 5 |
| Qun Chen et al, 1999 | 1998-1999 | Guangdong | Mix | 98 | Pen,Tet, Cip, Cef, Spe | 1 | 1 | 1 | 1 | 0 | 1 | 5 |
| Zhongwei Li et al, 1999 | 1995/4-1996/11 | Shandong | Patients | 204 | Pen,Tet, Cef, Spe | 0 | 1 | 1 | 0 | 1 | 1 | 4 |
| Hongru Zhao et al, 2001 | 1997 | Hebei | Patients | 69 | Pen,Tet, Cip, Cef | 1 | 1 | 0 | 0 | 1 | 1 | 4 |
| Zhisheng Wong et al, 2001 | 1997/7-1997/11 | Guangdong | Patients | 30 | Spe | 1 | 1 | 1 | 0 | 0 | 0 | 3 |
| Xiuqin Dai et al, 2001 | 1999 | Jiangsu | Patients | 112 | Pen, Cip, Cef, Spe, | 1 | 1 | 1 | 0 | 1 | 1 | 5 |
| Jingping Ge et al, 2001 | 1998/1-2000/1 | Jiangsu | Patients | 130 | Pen, Cip, Cef | 1 | 1 | 1 | 0 | 1 | 1 | 5 |
| Wei Li et al, 2001 | 1996-1999 | Guangxi | Patients | 221 | Pen,Spe, | 1 | 1 | 1 | 0 | 1 | 1 | 5 |
| Shui'e Zhang et al, 2002 | 2000/1-2001/12 | Hunan | Mix | 108 | Pen,Tet, Cip, Cef | 1 | 1 | 1 | 1 | 1 | 1 | 6 |
| Ying Cheng et al, 2002 | 2000/3-2000/12 | Shanghai | Patients | 225 | Pen, Cip, Cef | 1 | 1 | 1 | 0 | 1 | 1 | 5 |
| Yinhui Pei et al, 2002 | 2001 | Hebei | Patients | 155 | Pen,Tet, Cef | 1 | 1 | 1 | 0 | 1 | 0 | 4 |
| Xingzhong Wu et al, 2002 | 2001/1-2001/12 | Guangdong | Patients | 153 | Pen,Tet, Cip, Cef, Spe | 1 | 1 | 1 | 0 | 1 | 1 | 5 |
| Shengchun Wang et al, 2002 | 1993-2001 | Mix | Patients | 604 | Pen,Tet, Cip, Cef, Spe | 1 | 0 | 0 | 0 | 1 | 0 | 2 |
| Hong Li et al, 2003 | 2000/1-2002/10 | Henan | Patients | 98 | Tet, Cip, Cef, Spe | 1 | 1 | 1 | 0 | 0 | 1 | 4 |
| Shunzhang Ye et al, 2003 | 1993-2000 | Mix | Patients | 4976 | Pen,Tet, Cip, | 1 | 0 | 1 | 0 | 1 | 1 | 4 |
| Jianzhong Wang et al, 2003 | 2000/3-2001/3 | Guangdong | Patients | 173 | Pen,Tet, Cip, Cef, Spe | 1 | 1 | 1 | 0 | 1 | 1 | 5 |
| Bangyong Zhu et al,2003 | 2000/1-2001/11 | Guangxi | Patients | 160 | Pen,Tet, Cip, Cef, Spe | 0 | 1 | 1 | 0 | 1 | 1 | 4 |
| Yonghui Dong et al, 2003 | 2002/1-2002/10 | Xinjiang | Patients | 113 | Cip, Cef, Spe | 1 | 1 | 1 | 0 | 1 | 1 | 5 |
| Wenfei Li et al, 2003 | 2001/1-2002/6 | Shandong | Patients | 232 | Pen,Cip, Cef, Spe | 0 | 1 | 1 | 0 | 1 | 1 | 4 |
| Wenling Cao et al, 2003 | 1998/12-2002/11 | Guangdong | Patients | 603 | Pen, Cip, Cef, Spe | 1 | 1 | 1 | 0 | 1 | 1 | 5 |

^a^ Study population: patients (patients whose gender was not identified); men (men with urethritis); mix (male and female patients); women (women with endocervicitis)

**Abbreviations: PEN= penicillin; TET = tetracycline; CIP = ciprofloxacin; CEF= ceftriaxone; SPE= spectinomycin**
