# Supplementary material for: Antimicrobial resistance in Neisseria gonorrhoeae in China: a meta-analysis
Source: BMC Infect Dis. 2016 Mar 3;16:108. doi: 10.1186/s12879-016-1435-0 (PMC4778342; doi:10.1186/s12879-016-1435-0)
Supplement: Additional file 2: Table S2. — The included studies. (DOCX 23 kb) [file 12879_2016_1435_MOESM2_ESM.docx]

**Table S2 The included studies**

1. Zheng H, Yang B, Wu X et al. Antibiotic susceptibility of Neisseria gonorrhoeae isolates from Guangzhou, China, during 2002-2011. *Jpn J Infect Dis* 2014; **67**: 288-91.

2. Wu X. Comparison the in vitro anti-gonococal activities of spectinomycin and other four kinds of antibiotics. *Journal of Taishan Medical College* 2014: 287-9.

3. Wu W, Lao Le, Fang Z et al. Analysis of the antimicrobial resistance of Neisseria gonorrhoeae epidemic strains to seven antibiotics. *Diagnosis and Therapy Journal of Dermato-Venereology* 2014: 229-31.

4. Wenling Cao, Chao Bi, Yanhua Liang, Luyang Lin, Ping Li, Jiayan Li, Ping Huang, Xibao Zhang. Analysis of antibiotic resistance of Neisseria gonorrhoeae isolated from Guangzhou in 2013. p. 2, The Chinese Journal of Dermatovenereology.

5. Liu X, Shen S, Wei Q et al. The research on the susceptibility to antimicrobial and the resistance plasmids of Neisseria gonorrhoeae isolates in a certain area. *Laboratory Medicine and Clinic* 2014: 1530-1+4.

6. Li Y, Shi Z, Zhao P et al. The research on plasmid profiles of TEM-1 encoding gene in penicillinase-producing Neisseria gonorrhoeae and tetM gene in high level tetracyline-resistant Neisseria gonorrhoeae in Foshan. *Chinese Journal of Antibiotics* 2014; **39**: 236-9.

7. Huang H, Zhang H, Liang P. The analysis of antimicrobial susceptibility of 127 Neisseria Gonorrhoeae in a district from 2011~2012. *Guide of China Medicine* 2014: 12-3.

8. Guo C, Zhang H, Jiang M et al. Surveillance on antibiotic susceptibility and plasmid-mediated resistance of Neisseria gonorrhoeae in Panyu Guangzhou. *International Journal of Epidemiology and Infectious Disease* 2014; **41**: 160-3.

9. Zhang L, Mo J, Wang F et al. Surveillance on antibiotic susceptibility of Neisseria gonorrhoeae in Shenzhen From 2008 to 2011. *Chinese Journal of Preventive Medicine* 2013; **47**: 940-4.

10. Yu B, Gong Y, Huang H. Analysis of the drug‐resistance of Neisseria gonorrhoeae to six types of antibiotics in Huizhou. *Laboratory Medicine and Clinic* 2013: 1111-2.

11. Wu X, Huang J, Liu X et al. Antimicrobial resistance in 525 clinical isolates of Neisseria gonorrhoeae from 5 cities in Guangdong. *Diagnosis and Therapy Journal of Dermato-Venereology* 2013: 396-400.

12. Li L, Zheng Q. Analysis of the antimicrobial resisyance of Neisseria gonorrhoeae to six antibiotics. *Clinical Medicine* 2013: 113-4.

13. Jiang Y, Liu J, Xie Y et al. Study on drug resistance of 103 strains of Neisseria gonorrhoeae. *Practical Preventive Medicine* 2013: 345-7.

14. Gong Y, Yu B, Huang H. Research of Neisseria gonorrhoeae to antibiotics resisyance in Huizhou region. *China Modern Medicine* 2013: 167-9.

15. Chen W, Xie W. Surveillance on susceptibility of 204 Neisseria gonorrhoeae strains to 5 antimicrobial agents. *Jilin Medical Journal* 2013: 2051-2.

16. Wu X, Zheng H, Li Y et al. In vitro susceptibility to 6 antimicrobial agents and the resistance plasmids genotype of Neisseria gonorrhoeae isolates from 4 cities in Guangdong. *Diagnosis and Therapy Journal of Dermato-Venereology* 2012: 352-5+8.

17. Wu A. The analysis of antibiotic resistance of Neisseria gonorrhoeae in Jiangyan. *Chinese Journal of Misdiagnostics* 2012: 1119-20.

18. Li X, Cao W, Song W et al. Antibiotic resistance of Neisseria gonorrhoeae in Guangzhou: an analysis result. *Chinese Journal of Microecology* 2012: 554-5+60.

19. Lao Le, Fang Z, Wu W et al. In vitro susceptibility to 5 antinicrobial agents and TEM-1 genotypeing of clinical strains of Neisseria Gonorrhoea. *International Journal of Laboratory Medicine* 2012: 1801-2+5.

20. Liu X, Wu K, Wu X et al. Analysis of the drug-resisyance of Neisseria gonorrhoeae in Zhuhai area. *Diagnosis and Therapy Journal of Dermato-Venereology* 2011: 163-5.

21. Cao W, Li X, Bi C et al. Analysis of the drug-resisyance of Neisseria gonorrhoeae to 6 types of antibiotics in certain area. *International Journal of Laboratory Medicine* 2011: 2205-6.

22. Zhang L, He L, Wang F et al. Trend of drug resisyance of Neisseria gonorrhoeae in Shenzhen area in 2008. *China Tropical Medicine* 2010: 561-2.

23. Wu W. Analysis of antibiotics resistance of 128 strains of Neisseria gonorrhoeae. *Jilin Medical Journal* 2010: 1213.

24. Pei J, Zhang S, Huang J et al. Surveillance on susceptibility of 92 Neisseria gonorrhoeae strains to antimicrobial agents in Shantou Guangdong. *Diagnosis and Therapy Journal of Dermato-Venereology* 2010: 268-70.

25. Cai S, Sun S, Huang Q et al. Analysis of resistance of Neisseria gonorrhoeae in Shanguan area. *Journal of Clinical and Experimental Medicine* 2010: 1022-3.

26. Zhu X, Huang H, Ke J et al. Analysis of antibiotics resistance of Neisseria gonorrhoeae isolates in Jiangmen in 2008. *Journal of Guangdong Medical College* 2009: 639-41.

27. Pan J, Lin C, Lv W. Analysis of susceptibility of Neisseria gonorrhoeae to antibacterial drug. *Medical Laboratory Science and Clinics* 2009; **20**: 12-4.

28. Lin S, Chen G, Lai Z. Antibiotic resisyance of Neisseria gonorrhoeae in Shenzhen. *Chinese Journal of Public Health* 2009: 1096-7.

29. Li Y, Pan H, Wu X. Detection of the antibiotics susceptibe epidemic strains of Neisseria gonorrhoeae in Foshan city. *Journal of Tropical Medicine* 2009: 796-8.

30. Guo C, Li X, Guo Y et al. Analysis of antimicrobial susceptibility of Neisseria gonorrhoeae epidemic strains to five antibiotics. *International Journal of Laboratory Medicine* 2009: 566-7+70.

31. Cao W, Li X, Bi C et al. Analysis of antibiotic resistance of Neisseria gonorrhoeae in Guangzhou. *Chinese Journal of Microecology* 2009: 553-4.

32. Wu X, Zheng H, Zeng W et al. Surveillance of gonococcal antibiotic susceptibility in Giangzhou. *Diagnosis and Therapy Journal of Dermato-Venereology* 2008: 121-3.

33. Ren X, Shang H, Liu Er et al. Analysis of the resistance of Neisseria gonorrhoeae to 4 antibiotic in Shenzhen in 2005. *Shanxi Medical Journal* 2008: 307-8.

34. Ma Y, Song K, Wang H. A research on relationship between drug resistance to Neisseria gonorrhoeae in Shenzhen city ang plasmid of the bacterium. *Chinese Journal of Woman and Child Health Research* 2008: 314-6.

35. Wu X, Zheng H, Huang J et al. Analysis of antibiotic resistance of Neisseria gonorrhoeae in Guangzhou. *Chinese Journal of Infection and Chemotherapy* 2007: 215-8.

36. Wu X, Zheng H, Huang J et al. Surveillance on antibiotic susceptibility of Neisseria gonorrhoeae from 2000 to 2004 in Guangzhou. *Chinese Journal of Clinical Pharmacy* 2006: 277-9.

37. Cai W, Li X, Zhang X et al. Analysis of the resistance of Neisseria gonorrhoeae to 6 antibiotic in Guangzhou in 2005. *Journal of Tropical Medicine* 2006: 952-3+35.

38. Zhang X, Cao W. Investigation on the susceptibility and resistance of Neisseria gonorrhoeae to cefditoren in Guangzhou. *Chinese Journal of AIDS & STD* 2005: 290-1+318.

39. He Q, Tang X, Geng Q et al. The drug resisyance character variance of Neisseria gonorrhoeae in Shenzhen area during 2000 to 2004. *Chinese Journal of Birth Health & Heredity* 2005; **13**: 112-3.

40. Cao W, Li X, Bi C et al. Surveillance on 5 antibiotic resistance of Neisseria gonorrhoeae in Guangzhou in 2004. *Modern Preventive Medicine* 2005: 103-4.

41. Yang S, Wu Y, Yu M et al. The susceptibility of Neisseria gonorrhoeae to seven antibiotic agents. *Medical Science Journal of Central South China* 2004: 290-3.

42. Wu X, Zheng H, Huang J et al. Surverillance of resistance of Neisseria gonorrhoeae to 5 antibiotic agents. *Chinese Journal of Antibiotics* 2004: 570-3.

43. Wu X, Zheng H, Chen Y et al. Surverillance of resistance of Neisseria gonorrhoeae to ciprofloxacin. *Diagnosis and Therapy Journal of Dermato-Venereology* 2004; **11**: 122-3,6.

44. Cao W, Li X, Li J et al. Analysis of antibiltic resistance of Neisseria gonorrhoeae in Guangzhou 2003. *Chinese Journal of Microecology* 2004: 55+7.

45. Cao W, Li X, Li J et al. Analysis of five antibiltic resistance of Neisseria gonorrhoeae in Guangzhou. *Chinese Journal of Laboratory Medicine* 2004: 45-7.

46. Zheng HP, Cao WL, Wu XZ et al. Antimicrobial susceptibility of Neisseria gonorrhoeae strains isolated in Guangzhou, China, 1996-2001. *Sex Transm Infect* 2003; **79**: 399-402.

47. Wang J, Xu M, Cai X et al. Determination of antimicrobial susceptibility and plasmid profiles of Neisseria gonorrhoeae in Shantou. *China Journal of Leprosy and Skin Diseases* 2003: 8-10.

48. Cao W, Li J, Li X et al. Analysis of variation of neisseria gonorrhoeae resistance to five antibiotics in Guangzhou from 1998 to 2002. *Chinese Journal of Microecology* 2003: 48-9.

49. Wu X, Zheng L, Zheng H et al. Study on antibiotic resistant of Neisseria gonorrhoeae. *Diagnosis and Therapy Journal of Dermato-Venereology* 2002: 82-4.

50. Wong Z, Luo D, Chen X. The susceptibility text of Neisseria gonorrhoeae to spectinomycin. *Diagnosis and Therapy Journal of Dermato-Venereology* 2001; **15**: 37-8.

51. Wenling C, Xibao Z, Shi F et al. Analysis of the antibiotic sensitivity of Neisseria gonorrhoeae in Guangzhou, Peoples Republic of China. *Sex Transm Dis* 2000; **27**: 480-2.

52. Guoming L, Qun C, Shengchun W. Resistance of Neisseria gonorrhoeae epidemic strains to antibiotics: report of resistant isolates and surveillance in Zhanjiang, China: 1998 to 1999. *Sex Transm Dis* 2000; **27**: 115-8.

53. Chen Q, Li G, Wang S. Study on the Resistance of Neisser in Gonorrhoeae Epidemic Strains to Antibiotics. *Chinese Journal of Public Health* 1999: 75-7.

54. Zheng H, Zhang M, Liu Z et al. Analysis of Neisseria gonorrhoeae resistance to peneicillin and the Prevalence of PPNG nearly a decade. *Diagnosis and Therapy Journal of Dermato-Venereology* 1998: 7-8.

55. Zheng H, Liu Z, Zhang M et al. Analysis of the drug resistance of Neisseria gonorrhoeae to 5 antibiotics. *Chinese Journal of Antibiotics* 1998: 59-61+4.

56. Chen SC, Yin YP, Dai XQ et al. Antimicrobial resistance, genetic resistance determinants for ceftriaxone and molecular epidemiology of Neisseria gonorrhoeae isolates in Nanjing, China. *J Antimicrob Chemother* 2014; **69**: 2959-65.

57. Zhang C, Li Q. Analysis of antibiotic resistance of 60 Neisseria gonorrhoeae isolates. *Laboratory Medicine and Clinic* 2011: 843-4.

58. Yu Z. Analysis of the drug resistance of Neisseria gonorrhoeae in Ganyu area. *Chinese Journal of Clinical Laboratory Science* 2011: 304-5.

59. Niu Y. Analysis of the drug resistance of Neisseria gonorrhoeae to 5 antibiotics. *Laboratory Medicine and Clinic* 2011: 1580-1.

60. Dai X, Sun H, Yin Y. Analysis of antibiotic resistance of Neisseria gonorrhoeae isolates in Nanjing in 2007. *Chinese Journal of AIDS & STD* 2009: 183-4.

61. Su X, Jiang F, Qimuge et al. Surveillance of antimicrobial susceptibilities in Neisseria gonorrhoeae in Nanjing, China, 1999-2006. *Sex Transm Dis* 2007; **34**: 995-9.

62. Wang B, Xu JS, Wang CX et al. Antimicrobial susceptibility of Neisseria gonorrhoeae isolated in Jiangsu Province, China, with a focus on fluoroquinolone resistance. *J Med Microbiol* 2006; **55**: 1251-5.

63. Su X, Dai X, Sun H et al. Surveillance of antimicrobial susceptibilities of Neisseria gonorrhoeae in Nanjing city from 1999-2002. *Chinese Journal of Dermatology* 2004: 16-8.

64. Ge J, Ma H, Xue S et al. Analysis of antibiotic resistance of 130 Neisseria gonorrhoeae isolates in Nanjing. *Jiangsu Medical Journal* 2001; **27**: 932.

65. Dai X, Su X, Sun H et al. Analysis of antimicrobial suscepitibility of Neisseria Gonorrhoeae in Nanjing. *National Journal of Andrology* 2001; **7**: 200-1.

66. Xu Da, Wang Z. Analysis of antimicrobial suscepitibility of Neisseria Gonorrhoeae to 6 antibiotic agents. *Journal of Clinical Dermatology* 1997: 25-6.

67. Chu R, Meng W. Research on the Antibiotic Resistance and Plasmid Profiles of Prevail Neiossria Gonorrhoeae in Liaochen. *International Journal of Laboratory Medicine* 2011: 203-4+6.

68. Wu F. Study on antibiotic resistant of 135 Neisseria gonorrhoeae isolates. *Journal of Community Medicine* 2010: 45-6.

69. Pan K, Wei L, Hu S. The analysis on the antibiltics-resistance of clinical Neiossria Gonorrhoeae isolates in Jining area. *Journal of Pathogen Biology* 2008: 883+04.

70. Hou C, Liu Qa, Wu X et al. Study on The Antibiotuc Resistance and Plasmid Proffies of Neisseria Gonorrhoeae in Jinan. *The Chinese Journal of Dermatovenereology* 2008: 34-6.

71. Yang Y, Ni L, Han Z. Detection of antibiotic susceptibility of Neisseria Gonorrhoeae in Jining. *Chinese Journal of AIDS & STD* 2005: 48-9.

72. Li W, Ma J, Si X. Study on the susceptibility of Neisseria Gonorrhoeae to four antibiotic agents. *Preventive Medicine Tribune* 2003: 429-30.

73. Li Z, Yu M, Peng Y et al. Suscepbility of ten kinds of antibiotics against Neisseria Gonorrhoeae. *Chinese Journal of AIDS & STD* 1999: 29-30.

74. Wang X, Hou C, Liu Q et al. Research on antibiotic resistant of 165 Neisseria gonorrhoeae isolates. *China Journal of Leprosy and Skin Diseases* 2008: 114-6.

75. Zhu BY, Yu RX, Yin Y et al. Surveillance of antimicrobial susceptibilities of Neisseria gonorrhoeae in Nanning, China, 2000 to 2012. *Sex Transm Dis* 2014; **41**: 501-6.

76. Xie H, Zhu B, Li S. The analysis of antibiotic resistance in Neisseria Gonorrhoeae in Nanning fron 2009 to 2011. *The Chinese Journal of Dermatovenereology* 2012: 1008-9.

77. Xie J, Chen H. Surveillance of antimicrobial susceptibilities of 233 Neisseria gonorrhoeae isolates. *Medical Innovation of China* 2011: 121-2.

78. Tan D, Qin S, Li W et al. Surveillance of antimicrobial susceptibilities of 771 Neisseria gonorrhoeae isolates in Nanning. *Guangxi Medical Journal* 2010: 231-2.

79. Xie J, Chen H. Monitor on resistance of clinical isolated Neisseria Gonorrhoeae to ciprofloxacin in Nanning area between 1996-2008. *Chinese Journal of Infection Control* 2009: 277-9.

80. Zhu B, Zhao X. The analysis of antibiotic resistance in Neisseria Gonorrhoeae. *Journal of Clinical Dermatology* 2003: 199-200.

81. Li W, Zhao X, Qin S et al. Surveillance of susceptibility of 221 Neisseria gonorrhoeae isolates to penicillin and spectinomycin in Nanning. *Guangxi Medical Journal* 2001: 240-3.

82. Zhang TJ, Zhou XM, Zhang JL et al. Fluoroquinolone resistance among Neisseria gonorrhoeae isolates from Shanghai, China: Detection of quinolone resistance-determining region mutations. *Indian J Med Res* 2009; **129**: 701-6.

83. Yang Y, Liao M, Gu WM et al. Antimicrobial susceptibility and molecular determinants of quinolone resistance in Neisseria gonorrhoeae isolates from Shanghai. *J Antimicrob Chemother* 2006; **58**: 868-72.

84. Yang Y, Wu L, Gao Z et al. Surveillance of antimicrobial suscetibility of Neisseria Gonorrhoeae and its plasmid analysis in Shanghai from 2001 to 2003. *Laboratory Medicine* 2005: 293-6.

85. Cheng Y, Wang X, Quangui et al. Detection of antibiotic suscepitibility of Neisseria Gonorrhoeae in Shanghai, 2000. *Journal of Clinical Dermatology* 2002: 151-2.

86. Xu Q, Wang X, Zhang Y et al. Suscepbility of ten kinds of antibiotics against Neisseria Gonorrhoeae. *Journal of Clinical Dermatology* 1997: 16-9.

87. Gao Z, Wang X, Tang Q et al. Susceptibil ity of isolates of Neisseria gonorrhoeae to f ive antimicrobial agents. *Journal of Clinical Dermatology* 1997: 15-6.

88. Zhao X. Analysis of the resistance of Neisseria gonorrhoeae to 6 common antimicrobials in Hebei region. *Journal of Pathogen Biology* 2014: 637-41+46.

89. Gao H, Sun Y, Yang X et al. Analysis of the resistance of Neisseria gonorrhoeae to 6 antimicrobials in Tangshan. *Clinical Medicine of China* 2006: 518-9.

90. Pei Y, Ma L, Zhang S et al. Analysis of the antimicrobials resistance of Neisseria gonorrhoeae in Tangshan in 2001. *Journal of Hebei United University(Health Sciences)* 2002: 144-5.

91. Zhao H, Liu Y, Zhao C. Surveillance of antimicrobial suscetibility of Neisseria Gonorrhoeae. *Medical Research and Education* 2001: 38-9.

92. Zhong N, Liu Q, Qiao F et al. Antibiotic resistance surveillance in and genotyping of Neisseria gonorrhoeae isolates from Hainan province during 2011—2012. *Chinese Journal of Dermatology* 2014; **47**: 320-3.

93. Zhong N, Zheng Wa, Wang F et al. Analysis in the drug-resistance of Neisseria gonorrhoeae to antibiotics in Hainan area from 2006-2011. *The Chinese Journal of Dermatovenereology* 2013: 56-7+63.

94. Zhong N, Zheng Wa, Qiao F et al. Analysis of TEM-1 genotypeing of high-resistance tetracycline Neisseria Gonorrhoea in Hainan in 2011. *China Tropical Medicine* 2013: 1054-5+8.

95. Fang G, Liu X, Yuan Z. Analysis of antibiotic susceptinility of 266 Neisseria gonorrhoeae strains in Yueyang. *Journal of Clinical Research* 2007: 617-9.

96. Yang S, Wu Y, Yu M et al. Surveillance of antibiotic resistance of Neisseria gonorrhoeae in Hengyang. *Practical Preventive Medicine* 2005: 238-41.

97. Zhang Se, Zhou Q, Lie G. Analysis of the susceptinility of 108 Neisseria gonorrhoeae isolates to 5 antimicrobials. *Medical Science Journal of Central South China* 2002: 302-3.

98. Feng F, Ren Z, Sun M et al. Trend analysis of antibiotic resistance in Neisseria gonorrhoeae in Xi’an region, 2002—2009 *Chinese Journal of Dermatology* 2011; **44**: 591-2.

99. Ren X, Liu E, Shang X et al. Susceptibility of Neisseria gonorrhoeae to 4 antibiotics in 2006 in Shanxi area. *Evaluation and Analysis of Drug-Use in Hospitals of China* 2007: 359-61.

100. Feng L, Lin J, Yin A et al. Analysis of antibiotic resistance of 105 Neisseria gonorrhoeae strains in Xi’an. *Chinese Journal of Dermatology* 2005; **38**: 275.

101. Zhao J, Hu L, Gao J et al. Susceptibility of 118 Neisseria gonorrhoeae isolates. *Zhejiang Journal of Preventive Medicine* 2009; **21**: 32-,4.

102. Zhao J, Hu L, Lin F. Analysis of resistance of Neisseria gonorrhoeae from STD clinic patients. *China Journal of Leprosy and Skin Diseases* 2007; **23**: 422.

103. Gu K, Wu L, Zhu S et al. Analysis of 81 Neisseria gonorrhoeae isolates resistance to quinolones in Hefei. *Journal of Zhejiang University(Medical Sciences)* 1992: 164-6+77.

104. Zhou W, Yang S, Zhang S et al. Analysis of resistance of 87 Neisseria gonorrhoeae isolates to quinolones in Hefei. *Acta Universitatis Medicinalis Anhui* 2006: 81-2.

105. Xiong Z, Li T, Shen J et al. Detection of bacterial resistance in Neisseria gonorrhoeae *Chinese Journal of Microecology* 2005: 211-2.

106. ke D, Diao Q, Dai Xa et al. Surveillance of resistance of Neisseria gonorrhoeae to 5 antibiotic agents. *The Chinese Journal of Dermatovenereology* 2008: 549-50.

107. Qin Y, Zhu D. In vitro susceptibility to 4 antinicrobial agents Neisseria Gonorrhoea. *Journal of Chongqing Medical University* 1991: 114-7.

108. Xu K, Yu Q, Mo B et al. Analysis of Neisseria gonorrhoeae resistance in Nanchang during 2006 to 2008. *Journal of Nanchang University(Medical Sciences)* 2009: 134-5.

109. Li F, Yu K. Study on the antibiotic-resistance of Neisseria gonorrhoeae in Nanchang. *Journal of Nanchang University(Medical Sciences)* 2004: 37-8+41.

110. Wu B, Wang W, Li M. Analysis of Neisseria gonorrhoea susceptibility in Hohhot area. *Occupation and Health* 2007; **23**: 148-9.

111. Wang W, Wu B, Xu M et al. Comparison of antibiotic-resistance of Neisseria gonorrhoeae in Hohhot from two period. *Inner Mongolia Medical Journal* 2004: 1008-9.

112. Wang Z, Ren L, Zhuang W et al. Surveillance of Neisseria gonorrhoeae resistance in Fuzhou from 2001 to 2006. *China Journal of Leprosy and Skin Diseases* 2007: 1077-8.

113. Li X, Wang Z, Wang Z et al. Analysis of drug resistance of 98 strains of Nsisseria. *Chinese Journal of Health Laboratory Technology* 2003: 269-71.

114. Tu Y, Zhang L, Lin N et al. Surveillance on susceptibility of Neisseria gonorrhoeae to six antimicrobial agents in Wuhan. *China Journal of Leprosy and Skin Diseases* 2006: 451-2.

115. Wang X, Qu B, Tang R et al. Study of Neisseria gonorrhoeae antibiotic drug-sensitivity test. *Medical Journal of Chinese People＇s Health* 2006: 855+65.

116. Zhou X, Liu X, Zhang Z. Trend analysis of antibiotic resistance in Neisseria gonorrhoeae in Dalian from 2009 to 2011. *China health care and nutrition* 2013: 388-9.

117. Yong G, Yang X, Tan J et al. Surveillance of antimicrobial susceptibility of Neisseria gonorrhoeae in Chengdu, 2000-2004. *Chinese Journal of AIDS & STD* 2006; **12**: 242-4,20.

118. Dong Y, Zhi Q, Jin Y et al. Analysis of antimicrobial susceptibility of 113 Neisseria gonorrhoeae isolates in Urumqi in 2002. *Bulletin of Disease Control & Prevention(China)* 2003: 46-7.

119. Han Y, Yin Y, Dai X et al. Antimicrobial resistance in clinical isolates of Neisseria gonorrhoeae in China, 2008: an epidemiological study. *Chinese Journal of Dermatology* 2011; **44**: 472-5.

120. Dai X, Wang Z, Ren L et al. Analysis of susceptibility of Neisseria gonorrhoeae to spectinomycin. *China Journal of Leprosy and Skin Diseases* 2009; **25**: 77-.

121. Ye S, Wang Q, Su X et al. Epidemiological and bacteriological characteristics of Neisseria gonorrhoeae isolates in China. *Chinese Journal of Epidemiology* 2003: 41-4.

122. Wang S, Liu Y, Chen Q et al. Antimicrobial susceptibil ity and resistance correlation of Neisseria gonorrhoeae commonly used in China. *Negative* 2002: 1611-4.

123. Ye S, Su X, Wang Q et al. Surveillance of antibiotic susceptibility of Neisseria gonorrhoeae isolates fron ten cities of China from 1993-1998. *Chinese Journal of Dermatology* 2001: 12-4.

124. Ye S, Su X. Susceptibility of Neisseria Gonorrhoeae isolates to Spectinomycin. *Chinese Journal of AIDS & STD* 1997: 28-30.

125. Su X, Ye S. Antibiotic susceptibil ity of Neisseria gonorrhoeae to ciprofiloxacin. *Journal of Clinical Dermatology* 1997: 15-7.

126. Su X, Ye S, Chen P et al. Susceptibility of Isolates of Neisseria Gonorrhoeae to Five Antimicrobial Agents and Distribution of

Auxotype　*Chinese Journal of Dermatology* 1996: 20-2.

127. Ye SZ. Survey on antibiotic sensitivity of Neisseria gonorrhoeae strains isolated in China, 1987-1992. *Sex Transm Dis* 1994; **21**: 237-40.
