# Supplementary material for: Antimicrobial resistance in Neisseria gonorrhoeae in China: a meta-analysis
Source: BMC Infect Dis. 2016 Mar 3;16:108. doi: 10.1186/s12879-016-1435-0 (PMC4778342; doi:10.1186/s12879-016-1435-0)
Supplement: Additional file 3: Table S3. — Antimicrobial resistance rate of Neisseria Gonorrhoeae isolates. (DOCX 24 kb) [file 12879_2016_1435_MOESM3_ESM.docx]

**Table S3** **Antimicrobial resistance rate of Neisseria Gonorrhoeae isolates**

|  | Number of reports | Antimicrobial resistance rate of NG | |
| --- | --- | --- | --- |
|  |  | n/N | Percentage (95% CI) |
| **Penicillin** |  |  |  |
| Before 1995 | 25 | 2075/3187 | 58.1 (50.3,65.8) |
| 1996 | 6 | 825/1284 | 55.6 (40.5,70.7) |
| 1997 | 5 | 856/1691 | 59.6 (35.0,84.3) |
| 1998 | 4 | 885/1349 | 67.8(55.1,80.5) |
| 1999 | 8 | 1106/1599 | 62.9(47.5,78.3) |
| 2000 | 10 | 843/1089 | 74.4(64.1,84.7) |
| 2001 | 14 | 1494/1900 | 77.3(69.4,85.2) |
| 2002 | 8 | 753/911 | 81.3(74.3,88.2) |
| 2003 | 10 | 929/1194 | 81.4(74.0,88.9) |
| 2004 | 9 | 614/986 | 71.6(55.8,87.5) |
| 2005 | 7 | 591/856 | 87.3(78.9,95.6) |
| 2006 | 9 | 798/1080 | 81.5(73.4,89.5) |
| 2007 | 6 | 541/687 | 84.5(75.4,93.7) |
| 2008 | 7 | 688/757 | 90.5(86.0,94.9) |
| 2009 | 7 | 661/822 | 85.8(79.1,92.4) |
| 2010 | 4 | 349/530 | 66.0(56.6,75.5) |
| 2011 | 6 | 811/980 | 82.8(78.1,87.5) |
| After 2012 | 9 | 1684/2016 | 84.2(79.7,88.8) |
| **Tetracyclin** |  |  |  |
| Before 2000 | 17 | 2563/3648 | 68.3(58.7,78.0) |
| 2001 | 6 | 337/734 | 64.1(45.5,82.8) |
| 2003 | 6 | 5011/633 | 79.2(70.3,88.1) |
| 2004 | 3 | 156/282 | 65.7(30.4,93.1 |
| 2005 | 3 | 254/416 | 60.4(50.2,70.6) |
| 2006 | 4 | 465/576 | 76.8(58.5,95.0) |
| 2007 | 6 | 569/715 | 81.3(71.2,91.4) |
| 2008 | 7 | 779/840 | 91.7(86.4,97.0) |
| 2009 | 4 | 393/455 | 89.9(78.7,93.7) |
| 2010 | 3 | 335/394 | 86.7(78.8,94.6) |
| 2011 | 6 | 904/1097 | 79.7(71.1,88.3) |
| After 2012 | 8 | 1559/1822 | 71.3(33.6,82.4) |
| **Ciprofloxacin** |  |  |  |
| Before 1995 | 7 | 161/1244 | 12.7(08.6,16.7 |
| 1996 | 5 | 159/992 | 15.9(13.6,18.1) |
| 1997 | 5 | 416/1667 | 38.1(24.0,52.1) |
| 1998 | 6 | 932/1649 | 56.6(53.2,60.1) |
| 1999 | 11 | 1420/1976 | 77.3(73.5,81.2) |
| 2000 | 16 | 2090/2667 | 79.4(73.1,85.7) |
| 2001 | 17 | 1844/2198 | 83.1(79.1,87.2) |
| 2002 | 15 | 1511/1831 | 86.4(82.3,90.5) |
| 2003 | 19 | 2130/2359 | 93.8(91.9,95.7) |
| 2004 | 14 | 2262/2720 | 80.5(73.4,87.6) |
| 2005 | 11 | 1266/1337 | 96.9(95.4,98.5) |
| 2006 | 13 | 1176/1416 | 92.1(90.1,94.0) |
| 2007 | 14 | 1152/1298 | 96.0(94.5,97.4) |
| 2008 | 25 | 2465/2638 | 99.3(98.9,99.7) |
| 2009 | 12 | 1242/1346 | 95.0(92.8,97.3) |
| 2010 | 11 | 1207/1209 | 99.2(98.6,99.8) |
| 2011 | 10 | 1356/1467 | 93.9(91.0,96.9) |
| After 2012 | 10 | 1757/2036 | 96.3(95.1,97.4) |
| **Ceftriaxone** |  |  |  |
| Before 1995 | 6 | 30/1402 | 1.7(0.5,5.7) |
| 1996 | 5 | 1/1166 | 0.3(0.1,0.9) |
| 1997 | 4 | 9/1615 | 0.9(0.3,2.6) |
| 1998 | 3 | 0/1088 | 0.2(0.0,0.9) |
| 1999 | 9 | 39/1634 | 2.4(0.9,6.3) |
| 2000 | 13 | 18/2459 | 1.1(0.6,2.3) |
| 2001 | 17 | 16/1876 | 1.2(0.7,2.2) |
| 2002 | 13 | 3/1493 | 0.7(0.3,1.3) |
| 2003 | 15 | 6/2879 | 0.7(0.4,1.3) |
| 2004 | 12 | 22/1437 | 0.8(2.2,3.0) |
| 2005 | 12 | 4/1362 | 0.9(0.5,2.2) |
| 2006 | 16 | 21/1768 | 1.1(0.5,2.2) |
| 2007 | 12 | 7/1370 | 1.3(0.7,2.4) |
| 2008 | 24 | 14/2525 | 0.8(0.4,1.6) |
| 2009 | 12 | 1/13616 | 0.6(0.3,1.2) |
| 2010 | 11 | 1/1290 | 0.5(0.2,1.2) |
| 2011 | 10 | 8/1559 | 0.9(0.3,2.1) |
| After 2012 | 11 | 5/2143 | 0.5(0.2,1.4) |
| **Spectinomycin** |  |  |  |
| Before 1995 | 12 | 25/2056 | 1.4(0.5,3.0) |
| 1996 | 7 | 6/1374 | 0.8(0.4,1.6) |
| 1997 | 6 | 189/1721 | 1.4(0.4,4.4) |
| 1998 | 6 | 21/1676 | 1.4(0.3,5.8) |
| 1999 | 10 | 56/1846 | 0.9(0.3,2.9) |
| 2000 | 15 | 31/2567 | 1.4(1.0,2.0) |
| 2001 | 18 | 23/2283 | 1.2(0.7,1.9) |
| 2002 | 14 | 3/1816 | 0.6(0.3,1.2) |
| 2003 | 16 | 6/1834 | 0.7(0.4,1.3) |
| 2004 | 14 | 24/2716 | 0.8(0.3,2.0) |
| 2005 | 10 | 6/1168 | 1.0(0.5,1.8) |
| 2006 | 16 | 28/1745 | 1.6(1.0,2.5) |
| 2007 | 13 | 21/1740 | 0.9(0.4,1.7) |
| 2008 | 26 | 106/2712 | 1.0(0.6,1.7) |
| 2009 | 12 | 6/1346 | 0.6(0.3,1.2) |
| 2010 | 10 | 0/1185 | 0.5(0.2,1.1) |
| 2011 | 10 | 0/1452 | 0.6(0.3,1.2) |
| After 2012 | 11 | 3/2248 | 0.3(0.2,0.8) |
