# Supplementary material for: Antimicrobial resistance in Neisseria gonorrhoeae in China: a meta-analysis
Source: BMC Infect Dis. 2016 Mar 3;16:108. doi: 10.1186/s12879-016-1435-0 (PMC4778342; doi:10.1186/s12879-016-1435-0)
Supplement: Additional file 4: Table S4. — The Begg and Egger test of heterogeneity. (DOCX 14 kb) [file 12879_2016_1435_MOESM4_ESM.docx]

**Table S4 The Begg and Egger test of heterogeneity**

| Antimicrobial | No. of Study | No. of Reports | Resistance | | *Z* (Begg's test) | *P* | t (Egger's test) | *P* |
| --- | --- | --- | --- | --- | --- | --- | --- | --- |
|  |  |  | *I^2^* | *P* |  |  |  |  |
| PEN | 91 | 154 | 95.16% | 0.001 | 5.24 | 0.001 | 5.47 | 0.001 |
| TET | 62 | 73 | 95.80% | 0.001 | 4.59 | 0.001 | 5.31 | 0.001 |
| CIP | 113 | 221 | 96.91% | 0.001 | 3.70 | 0.001 | 9.50 | 0.001 |
| CEF | 103 | 205 | 64.89% | 0.001 | 5.94 | 0.001 | 5.94 | 0.001 |
| SPE | 113 | 226 | 74.62.% | 0.001 | 9.68 | 0.001 | 25.70 | 0.001 |

**Abbreviations: PEN= penicillin; TET = tetracycline; CIP = ciprofloxacin; CEF= ceftriaxone; SPE= spectinomycin**
